# Effect of Noninvasive Static Human Data on Maximum Data in Exercise

**DOI:** 10.3390/ijerph20021612

**Published:** 2023-01-16

**Authors:** Yichen Wu, Yining Sun

**Affiliations:** 1 Anhui Province Key Laboratory of Medical Physics and Technology, Institute of Intelligent Machines, Hefei Institutes of Physical Science, Chinese Academy of Sciences, Hefei 230031, China; 2 Science Island Branch of Graduate School, University of Science and Technology of China, Hefei 230031, China; 3 School of Electronic and Information Engineering, Anhui Jianzhu University, Hefei 230601, China

**Keywords:** maximum data in exercise, maximum heart rate, peak oxygen uptake, maximum power, body composition, exercise intensity

## Abstract

Maximum data in exercise (Max-Ex), including maximum heart rate (HR_max_), peak oxygen uptake (VO_2pk_), maximum power (MaxP), etc., are frequently used, whether it is for the determination of exercise intensity, the measurement of an athlete’s performance, assessment of recovery from disease, and so on. However, very often this choice does not take into account the targeted individual. We recruited 32 males and 29 females to undergo an incremental graded exercise test (GXT). Therefore, our study seeks to determine variations in Max-Ex, according to the noninvasive static human data (Non-In data). Data showed a significant relationship (*p* < 0.001) between body composition and Max-Ex. Of the 41 types of Non-In data we collected in communities, the body composition generally showed high correlation (maximum r = 0.839). 57.5% of the data, of which r > 0.6 were about body composition. The muscle-related body composition data had a greater effect on power, and the fat-related ones had a greater effect on HR_max_ and VO_2pk_. For some types of Max-Ex, the older and younger ones showed specific differences. Therefore, these results can be employed to adequately prescribe personalized health promotion programs according to diversity and availability, and have some reference value for other studies using Max-Ex.

## 1. Introduction

In today’s era, the biology of the human body is increasingly at risk due to a lack of physical activity and its consequences, particularly resulting in overweight and obese conditions. Sedentary behavior and physical inactivity are among the leading modifiable risk factors worldwide for cardiovascular disease and all-cause mortality. The World Health Organization classifies physical inactivity as the fourth leading cause of global mortality and the primary cause of many chronic disorders. It is estimated that the annual global health cost of low levels of physical activity exceeds USD 67 billion, and that sedentary lifestyles cause around 5 million deaths per year [1]. In an analysis based on data from 122 countries, more than 31% of adolescents and adults over the age of 15 were physically inactive [2]. Inactivity is more common in wealthy countries and among women and older people, and it is a factor in the development of noncommunicable diseases [3]. The promotion of physical activity and exercise training leading to improved levels of cardiorespiratory fitness is needed in all age groups, races, and ethnicities, as well as both sexes, to prevent many chronic diseases, especially cardiovascular disease [4].

“Exercise is also a good medicine”, and this statement has been proven in many studies. Leddy et al. [5] found that early treatment with subsymptom threshold aerobic exercise safely speeds recovery from sport-related concussion and reduces the risk of persistent postconcussive symptoms. Benedetti et al. [6] suggest that physical exercise is an effective means to stimulate bone osteogenesis in osteoporotic patients. In the study by Yang et al. [7], physical exercise was one of the therapeutic tools for atherosclerosis.

We all know that exercise is good for health, but it is worthwhile to study exactly what indicators are affected by exercise and what kind of effect it has on the human body. For human data during exercise, the maximum data obtained under individual exhaustion are relatively more meaningful. Lakoski et al. [8] found that the important factors related to cardiorespiratory function (most are expressed using Max-Ex data) are BMI, age, etc., accounting for 56% of the variation. For the participants with a similar level of physical activity, those with a normal BMI had a higher cardiorespiratory function compared to obese persons. The obesity may negate the benefits of physical activity, even in a healthy population of men and women. However, their study involved relatively few dimensions of non-exercise human data, and the analysis of the effects of exercise on these internal indicators may be somewhat lacking. Faude et al. [9] studied the pattern of changes in blood lactate concentration in humans during graded exercise experiments from small to maximum exercise intensity, clarifying the significance of aerobic and anaerobic thresholds. Zanini et al. [10] studied from an intracellular perspective the effects of mitochondrial DNA on exercise, including exercise performance, exercise injury, and immune competence. However, blood lactate concentrations and mitochondrial DNA are data that require invasive manipulation in specialist laboratories to obtain and are difficult to generalize to the general health promotion field. This study therefore aims to analyze its impact on in-exercise data, particularly the more important maximum data, through multidimensional noninvasive human data that can be collected based on the community health promotion system.

Further, the human motion that we see from a macro point of view is actually the result of the joint action of multiple internal systems in the body at the micro level. Therefore, studying the effects of static human body data on these exercise Max-Ex data is needed. For exercise intensity, most of them are anchored by the Max-Ex data to determine intensity, so the interrelationship of Non-In and Max-Ex data helps medical professionals to calibrate personalized exercise prescriptions, and for scientific studies, it has some interpretative reference value for the results obtained by academics.

All of our static data come from our independently developed Health Promotion Community Service System, which combines health assessment, personalized prescription (prescribing, implementation, and monitoring), and other functions. More than ten service stations have used the system in different communities, and nearly 10,000 people have experienced it. In terms of ease of use and accessibility, the system captures noninvasive data that are easy to collect. So, understanding the intrinsic link between these multidimensional noninvasive data and the Max-Ex points is a current need, both for personalized prescribing and other scientific research.

## 2. Materials and Methods

### 2.1. Participant

Sixty-one participants (32 males and 29 females) were recruited to the Institute of Intelligent Machines, Chinese Academy of Sciences, Hefei, China to participate in the experiment through advertisements on community health promotion stations and social media (smartphone applications). They all signed an informed consent form prior to the experiment. The age of the participants ranged from 23 years old to 63 years old (aged 20–29, 11 females and 12 males; aged 30–39, 6 females and 9 males; aged 40–49, 5 females and 8 males; aged 50–59, 7 females and 2 males; aged 60–69, 1 male), and the questionnaire revealed that there was a variation in lifestyle and exercise habits. We also confirmed that they had no contraindications to exercise before the trial.

### 2.2. Experimental Procedures

The experiment was scheduled to take place at least two hours after a meal (10:00 to 12:00 in the morning or 2:30 to 4:30 in the afternoon), maintaining the same ambient temperature (25 °C) and trying to maintain a static rest state between a meal and the start of the test. In addition, participants were asked not to drink caffeine, alcohol, or any other stimulants and not to perform any type of physical exercise for 24 h prior to the experiment. Before the start of the exercise trial, participants adjusted the seat and handlebar position to suit their body shape to ensure a comfortable ride. It is generally recommended that the distance from the seat to the lowest point of the pedals ensures that the participant’s entire leg is fully extended. Prior to the test, participants were familiarized for 3 min with the testing procedures and exercise technique, which served as a warm-up before the test. Participants also received standardized instructions on the Borg rating of perceived exertion (RPE) 6–20 scale [11]. All of the above procedures were implemented in order to standardize data collection for all participants. We used the bicycle ergometer (manufacturer: Lode BV Medical Technology, the Netherlands. IEC 60601-1. REF no. 960912) to perform graded exercises with increasing load power, where each level of exercise has a duration and interval of three minutes. We continuously monitored multidimensional exercise data (oxygen uptake (VO_2_), ventilation, heart rate (HR), ECG, etc.) throughout, both for exercise and interval periods, where the different levels of exercise load based on the bicycle are set at 20%, 40%, 50%, 70%, 85%, and 100% of the maximum load [12]. In the final stage of each level, the VO_2_ values were averaged every 5 s and the highest mean value obtained was taken as the final VO_2_ of the level, while the final HR of the level was taken as the highest HR value observed during the test. If a participant was unable to complete the 3 min test, the data could not be used until the heart rate and oxygen uptake had stabilized, and data that could not reach this stabilization were discarded. The HR and VO_2_ collected within the load level completed by the participant at exhaustion were used as the HR_max_ and VO_2peak_. Then, RPE as measured at the final stage of the 3 min exercise test at each level. Each participant’s rest heart rate (RHR) (wattage is 0, 0% of W-max) was used with the heart rate and power data of each level to construct a linear regression model of heart rate and wattage and to obtain the slope S_h_ of the graph of the linear model. We also used the same method to obtain the slope S_o_ of oxygen uptake and wattage. However, it is important to note that the oxygen uptake used here is the VO_2_ per kilogram of weight.

### 2.3. Measured Features and Statistical Analysis

Before the experiment, the Non-In data were collected for each participant. Exercise data were collected during and after the completion of the experiment for each participant. Pearson correlation analysis on the exercise data was performed for all quantifiable features (all Non-In data in Table 1). All participants were then divided into two groups using the age cut-off of 40 years (young set and old set) and then subjected to separate correlation analyses. The test presenting the best fit for each analysis was adopted, and r values were classified according to the recommendations from Safrit and Wood [13], i.e., 0–0.19 as no correlation, 0.20–0.39 as low correlation, 0.4–0.59 as moderate correlation, 0.6–0.79 as moderately high correlation, and 0.8–1.0 as high correlation.

## 3. Results

All participants are guided to complete as high a load level as possible and to complete the RPE test. However, it is possible that due to the level of subjective coordination or other unknown subjective and objective factors, only 80% of participants completed at least five levels of the GXT (with 28% completing level 6).

Direct exercise data (HR_max_, VO_2pk_, MaxP, reserve heart rate (RvHR), reserve oxygen uptake (RvVO_2_)) and indirect exercise data (S_o_ and S_h_) were collected during the experiment. For the purpose of multiperspective analysis, we divided the participants into three data sets: all, over 40, and under 40 years old. The data are statistically different after nonparametric testing (*p* < 0.05) in both direct and indirect exercise data.

### 3.1. Linear Regression Model of Heart Rate and Wattage and Oxygen Uptake and Wattage

The data of different load levels and RHR/rest oxygen uptake (RVO_2_) were used one-by-one to construct a linear regression model for each participant. For each participant’s data, one single level of data was randomly selected as the test set, and the rest of the levels were used as the training set for the model. The average R^2^ value of all the individuals’ models of HR and wattage was 0.979, the standard deviation was 0.019, and the average *p*-value was 5.64 × 10^−4^. The average R^2^ value of the models of VO_2_ and wattage was 0.980, the standard deviation was 0.015, and the average *p*-value was 6.83 × 10^−4^. This shows that the linear regression model has high prediction accuracy, so the use of the univariate slope S_h_ and S_o_ can describe the graphic characteristic of the model well. Then, we included all levels of data (regardless of training or test set) to reconstruct the model for each person. The slope S_h_ and S_o_ of the model’s graph was obtained from each participant (the mean value, standard deviation, median, the maximum and minimum of S_h_ and S_o_ are 0.684 and 0.167; 0.139 and 0.037; 0.678 and 0.161; 0.950 and 0.248; 0.441 and 0.094). The S_h_ and S_o_ are indirectly derived data compared to several other exercise data.

### 3.2. Correlation Results between Exercise Data and Noninvasive Static Human Data

**Figure 1:**
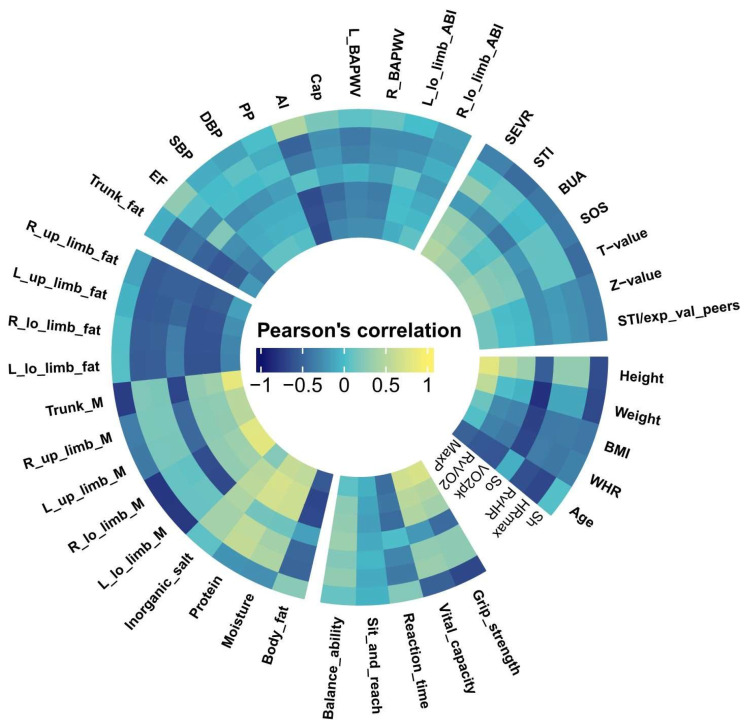
Relationships of all Non-In data with seven exercise data in cyclic heat map. The correlation data represented by each sector in the circle correspond to Table 1 and are divided into five main categories in the same way. Some variable names have been abbreviated for graphic aesthetics. R_, Right; L_, Left; lo_, lower; up_, upper; M, muscle; exp_val_peers, expected value of peers.

**Figure 2:**
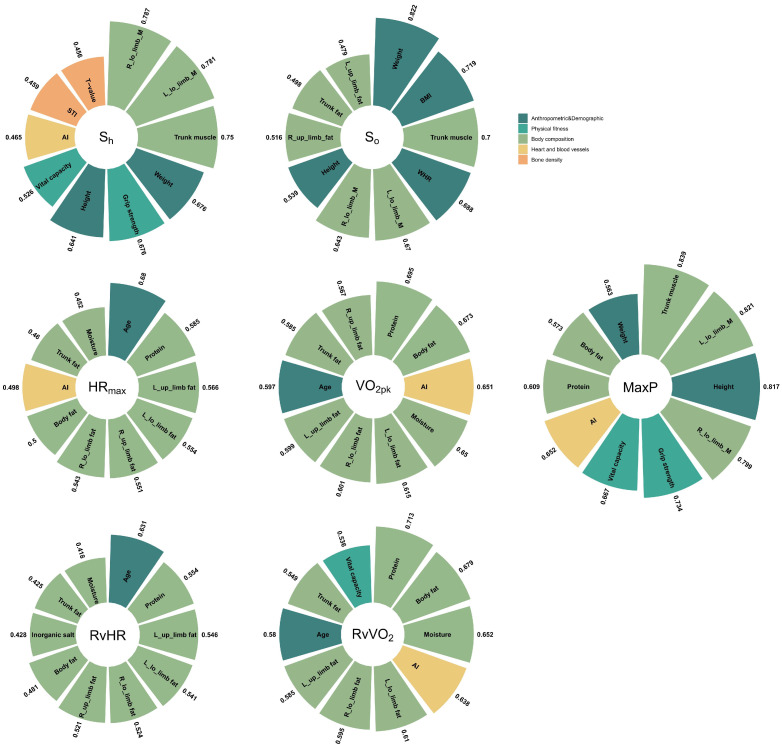
Details of the top ten Non-In data with the relationships to each of the exercise data. The different colors represent the specific categories to which the data belong. The numbers outside the sector represent their correlation values.

**Figure 3:**
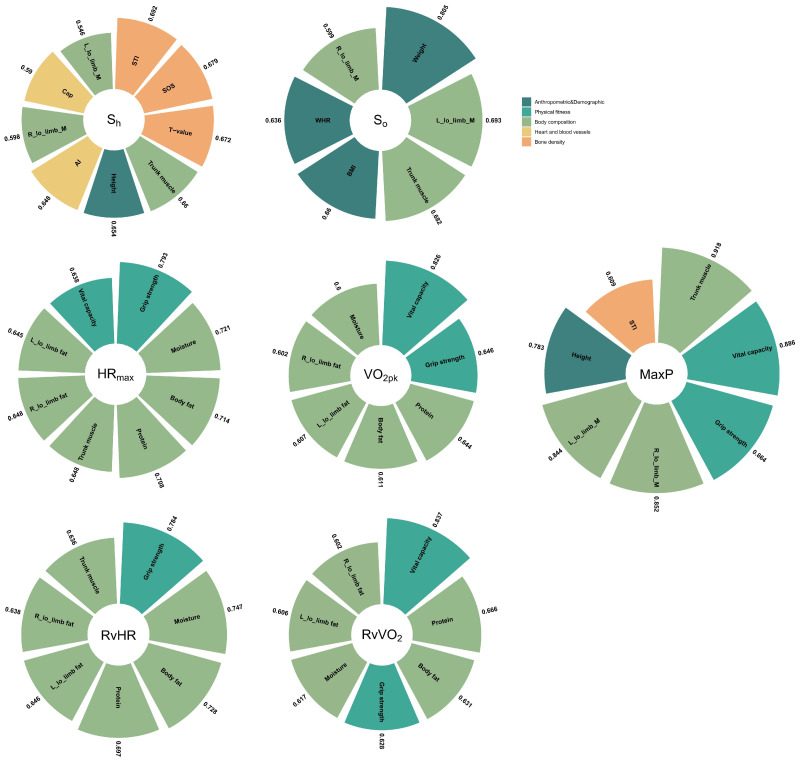
Details of the Non-In data with the relationships (*p* < 0.001) to each of the exercise data for the participants older than 40. The different colors represent the specific categories to which the data belong. The numbers outside the sector represent their correlation values.

**Figure 4:**
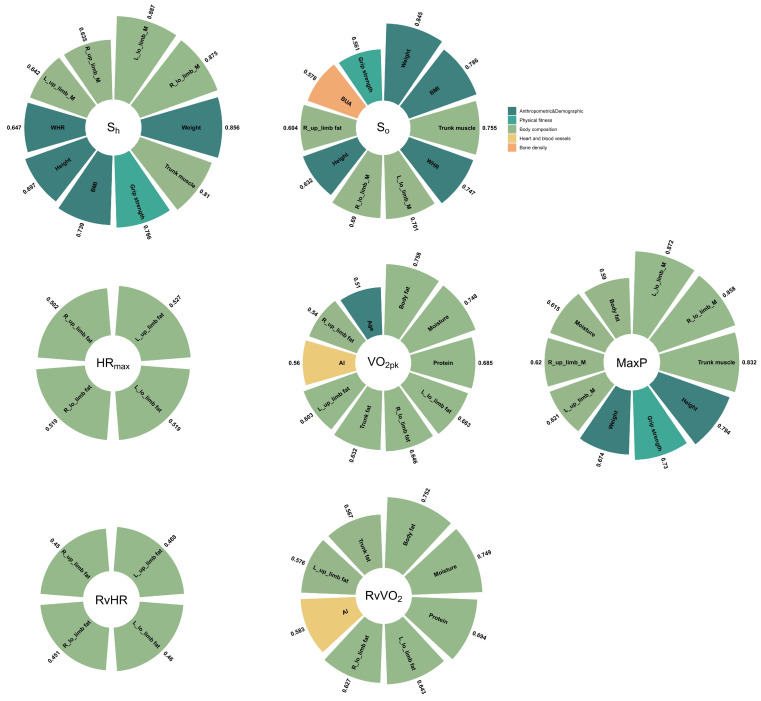
Details of the Non-In data with the relationships (*p* < 0.001) to each of the exercise data for the participants younger than 40. The different colors represent the specific categories to which the data belong. The numbers outside the sector represent their correlation values.

**Figure 5:**
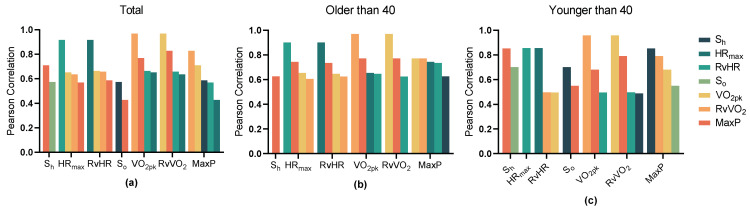
Details of the relationship between all types of data in exercise data with the significant relationships (*p* < 0.001) and divided into three sets based on age. (**a**) Total; (**b**) Older than 40; (**c**) Younger than 40.

The relationships (*p* < 0.001) were observed between the exercise data and Non-In data for all participants (Figure 1). Moreover, the top ten Non-In data with the relationships to each of the exercise data (*p* < 0.001) for all participants are shown in Figure 2. Figure 3 and Figure 4 show the same type of data as Figure 2, but for a different participant set (old and young). There are fewer Non-In data with relationships to exercise data (*p* < 0.001) in the two different age subsets, so it is not possible to present the top ten data as in Figure 2. Lastly, the relationships between the exercise data are shown in Figure 5.

## 4. Discussion

The main findings of the research were the correlations observed between multidimensional Non-In data and the specific exercise data obtained during an GXT using the exercise of a bicycle ergometer by the participant. It would be valuable to refer to these correlations from a variety of perspectives, whether for exercise prescription, assessment of exercise capacity, or other exercise-related study.

The Non-In data with significant correlation to the exercise data are mostly concentrated in the body composition and anthropometric and demographic categories (see Figure 1). The 81.81% of the Non-In data with moderately high or high correlation (r > 0.6) fall into the two categories above, and 33% of those are directly related to muscle, 18% are directly related to fat, and many of the rest are also indirectly related to them (see Figure 2).

VO_2pk_ represents the highest rate at which oxygen can be transported and used during aerobic exercise. It is recognized as the best single criterion of aerobic fitness [14] and is considered the gold standard for controlling exercise intensity during aerobic training programs. Our analysis shows that the largest effect on VO_2pk_ was mainly exerted by the body composition (especially fat-related data), followed by AI (reflection of heart function) and age, but there are few differences between younger and older people (see Figure 2, Figure 3 and Figure 4).

Our study further found that in addition to body composition data, vital capacity and grip strength were the data that showed correlations in the old set (r = 0.826, 0.646), as vital capacity is one measure of oxygen metabolism [15] and grip strength is one measure of muscle strength [16]. Furthermore, the correlations of the data for the body composition in old set are all somewhat reduced compared to the total set. In contrast, for the young set, the correlation of the data for the body composition was significantly higher. For data with correlations in both the young- and old-aged sets (see Figure 3 and Figure 4, body fat, muscle, water, L_low_limb_fat, L_low_limb_fat), the standard deviations of the correlation coefficients in the young set are all smaller than those in the old sample. This greater individual variation (SD become larger) may be due to lifestyle changes in older people, an increase in underlying diseases, and ageing of the musculoskeletal system [17], etc., some of which have already been argued and some of which require continued research. The RvVO_2_ takes into account the resting oxygen uptake, so the correlations of the Non-In data that have an effect on it are all somewhat stronger than VO_2pk_.

We can see that the dataset with high correlation with HR_max_ obtained based on the total set is very similar to that of VO_2pk_. This may be due to the fact that HR_max_ and VO_2pk_ are themselves directly strongly correlated [18]. However, the correlation coefficient for HR_max_ was then slightly lower than those for VO_2pk_, except for the age factor (see Figure 2). Compared to VO_2pk_, HR_max_ is clearly influenced by age, and as age becomes younger, the effect of body composition data on HR_max_ becomes so small that it may no longer be of much reference value (see Figure 3 and Figure 4). This means that in the young set, the multidimensional data that we have obtained so far have not been found to have a significant effect on HR_max_. However, in the old set, body composition data, vital capacity, and grip strength continue to be the ones that have a large effect on HR_max_.

In particular, the Non-In data that we obtained as having an effect on Max power had a highest correlation coefficient than the other exercise data. (r = 0.839, 0.821, 0.817, 0.799 correspond to trunk muscle, left lower limb muscle, height, and right lower limb muscle, respectively; see Figure 2). Moreover, it is also the highest in both the young and old set. Especially for the old set, the highest correlation exceeded 0.9, and similarly to VO_2pk_ and HR_max_, vital capacity and grip strength show a strong correlation, and notably, the STI data show an influence that is similar to previous studies [19,20]. Also different is the fact that among young people, grip strength shows a stronger correlation for the first time. The details of the body composition will certainly vary depending on the exercise being tested; in our case, it was the bicycle exercise where the lower limb muscles were more involved, so the correlation was relatively high.

The effects of physical exercise on health are dose-dependent [21]. Many scholars have more or less shown in the research that too much exercise is as bad as too little. Unproper overexercise might also cause damage to health. Conversely, the benefits of exercise can be difficult to achieve with too little exercise. Therefore, determining exercise intensity is key, and is one of the core steps in generating personalized exercise prescriptions [22].

Prescribing exercise intensity relative to Max-Ex, which is to develop a percentage of the individual’s maximum data to determine intensity, is one of the most commonly used methods. VO_2pk_, HR_max_, and MaxP also often represent the highest exercise intensity, which is then used as a maximum anchor point [23,24]. A professional or intelligent system will develop an exercise prescription based on knowledge and experience; for example, the recommended exercise program for an individual’s exercise prescription is outdoor running, with an exercise intensity of 70% of HR_max_ for a duration of 20 min, three times per week. The maximum data are one of the keys to the accuracy of the exercise prescription. However, the Max-Ex (especially for VO_2pk_) requires very stringent conditions to be obtained, and therefore, its use outside the laboratory is limited. Especially in the field of health promotion for the general public, both VO_2pk_ and HR_max_ are also often calculated using specific formulas, with VO_2pk_ being based on a specific type of exercise [25] and HR_max_ being based on age [26]. Although the formulae are simple to use, they may yield extrapolated values that are too high or too low compared to the actual measured values [27]. Subsequent improved formulas have been developed, but they also tend to apply only to populations similar to those for which they were derived. This falls short of our goal of achieving personalized exercise recommendations in the area of public health promotion.

The difference in correlation shown by the Non-In data (mainly body composition) for Max-Ex (VO_2pk_, HR_max_, etc.) in our study therefore makes it desirable to develop and implement intervention programs for physical activity for different individuals. Take HR_max_ as an example; the existing formula is mainly calculated by age, and the professional can increase or decrease the HR_max_ obtained from the formula according to the difference between the individual’s Non-In data, which are related to the HR_max_ and young people or the difference with the same age. The degree of calibration currently relies mainly on professionals, so the creation of relevant models by computer is one of our later tasks. Moreover, as these Non-In data are themselves sourced from community health promotion service stations, the results of the study can then be more easily applied to the people in the community.

On the other hand, the Max-Ex data are a key indicator of great interest in many scientific studies. The changes in Max-Ex data are a common indicator of the degree of recovery of specific bodily functions after the treatment of many diseases [28], a way to study side effects of drugs [29], a common indicator of the effectiveness of different types of training [30], and a commonly used measure of an athlete’s athletic ability [31]. For example, in the case of postcure assessment, there may be small errors when measured simply by Max-Ex data. Long periods of bed rest can lead to changes in data such as body composition. Even the absence of disease may lead to a change in the maximum value. Similarly, evaluating the effectiveness of a training modality through maximum data can lead to some bias if the differences in individual Non-In data are not taken into account. Even if the actual training is less effective, Max-Ex data may be improved if other factors (diet or lifestyle) change the individual body composition and other Non-In data that can influence the Max-Ex data. Furthermore, professionals can adjust the assessment results according to our findings by excluding confounding factors and selecting controllable variables.

For S_h_ and S_o_, the correlation coefficients for Non-In data obtained from the young set were the highest overall, followed by the total set and the old set, in that order. The Non-In data with high correlation coefficients are also concentrated in the body composition and anthropometric and demographic data. However, for S_o_, especially in the old set, the type of Non-In data with higher correlations becomes relatively more varied, with the addition of the categories of bone density and heart and blood vessels.

In addition, S_h_ and S_o_ are obtained indirectly by constructing our computational model. A high linear correlation was observed between heart rate and wattage or oxygen uptake and wattage, which average R^2^ by fitting a linear model: 0.979 and 0.980 for S_h_ and S_o_, respectively. If the few individuals with low-power-level completion are excluded, the average R^2^ will be even higher. Such findings are in agreement to those reported by a previous researcher [32]. Some researchers have also found that this relationship shifts from linear to curvilinear during the latter stages of a GXT [33].

However, only a very small proportion (less than 10%) of the people in our study have this phenomenon. The reason for this result may be that on the one hand, our sample size is relatively larger and includes a relatively wider range of people, including young, middle-aged, and older people, most of whom do not have regular exercise habits [34], and the economy of exercise was varied [35]. On the other hand, due to the large number of participants, there are still some whose motivation in the test may not be guaranteed. Even in these few participants, the difference in average R^2^ obtained after fitting the curvilinear and linear models separately is very small (mean value of the difference is 0.011). Experimental error may also contribute to this difference, although we know from the RPE data that 97% of the participants reached exhaustion (over scale 18) and only 9.8% of participants had an HR_max_ obtained in the test that differed from the HR_max_ calculated by the formula [26] by more than 30 bpm. For most ordinary people, they are exhausted and can no longer continue before their work rate (HR vs. wattage, VO_2_ vs. wattage) image enters the curved section. Linear models are simpler to construct and easier to use than curvilinear models. Therefore, in the field of health promotion for the general public, where convenience, generalizability, applicability, etc., are valued, such as to determine exercise intensity, linear models can be widely used instead of curvilinear models to describe work rate.

Interestingly, we found that most of the body composition data with high correlations to power (max power) and power-related data (S_h_, S_o_) were about muscles (trunk and limb muscles), while most of the body composition data with high correlations to other exercise data (VO_2pk_, HR_max_, RvVO_2_, RvHR) were about fat. This may be due to the fact that performing work (power) is a conversion of energy by the body to the outside world through exercise (the body consumes heat and converts it into external mechanical energy), while VO_2pk_, HR_max_, etc., are intrabody indicators of the body in exercise, but this still requires subsequent research. Of course, the significance of such results may also be limited because our sample was limited, with only two obese individuals (BIM ≥ 30) and three lean individuals (BMI ≤ 18.4) in our experimental population, and all just over the threshold.

VO_2_ is considered the gold standard for controlling exercise intensity during aerobic training programs, and our research also indirectly shows this. In Figure 5a, we can see that the highest correlations with maximum power are for RvVO_2_ (r = 0.829) and VO_2pk_ (r = 0.769), respectively, and there is no significant difference between younger and older people (see Figure 5b,c). This suggests that of the exercise data, it is the ability to exchange oxygen that has the greatest effect on a person’s ability to perform work. However, the high correlation does not imply “cause and effect”. We also found that some people instead had a smaller exercise max power than those whose VO_2pk_ or HR_max_ was smaller than theirs. However, to simply dismiss a high correlation between two variables having high construct validity might result in an investigator missing an important point. Therefore, when assessing a person’s cardiorespiratory capacity or aerobic capacity, would we not be better off combining S_h_ and HR_max_ or S_o_ and VO_2pk_ instead of passing a single Max-Ex? This is something we need to follow up on. Then, the indirect exercise data, S_h_ and S_o_, are less correlated with other intrabody indicators in exercise, and are mainly influenced by power, as well as by each other. In terms of the sample set, the highest consistency (the correlation of exercise data with each other) of exercise data was found in the old sample and the lowest in the young sample.

A limitation of the present study is that our experiment is based on cycling, and although it is a common way of experimenting with GXT, it is not clear whether the different forms of exercise have any effect on the results we obtain. Moreover, the individual’s exercise technique directly affects the economy of movement, which may ultimately have an impact on the outcome. Additionally, the sample size may also be mentioned as a possible limitation. The use of 40 years as the basis for the division between young and old is not very scientific, mainly because we did not have enough older people (older than 50) to participate in the experiment. On the other hand, the type of Non-In data we collect is not specifically designed for this purpose, but mainly comes from the dimensions that are already available in our existing set of health monitoring and promotion systems, which are mainly used in the community and serve the general public, and the data collected are noninvasive. There is a relative lack of indicators concerning the cardiopulmonary system, with some blood indicators (blood oxygen, lactate threshold, etc.) and cell molecular data (mitochondria, etc.) missing. Accordingly, we suggest that future studies extend and compare more dimensional parameters and investigate them in larger and/or different populations, as well as using different exercises to confirm and expand our findings.

## 5. Conclusions

In conclusion, this study collected participants’ Max-Ex data and more than forty dimensions of easily accessible Non-In data for analysis. The findings showed high correlations with Max-Ex data in our multidimensional Non-In data, with data on body composition type having the greatest effect, especially those of them directly or indirectly linked to muscle and fat, and with some differences in age. Based on these results, professionals can better develop exercise prescriptions based on individual differences and availability. Furthermore, the relationships obtained between the Max-Ex and Non-In data will have some interpretative reference value for other studies that use them.

## Figures and Tables

**Table 1:** Static human body data for the participants according to sex. Values refer to mean ± standard deviation (SD).

Data Type	Feature	Females (n = 29)	Males (n = 32)	Data Type	Feature	Females (n = 29)	Males (n = 32)
Anthropometric and demographic	Height, cm	161.1 ± 4.68	172.4 ± 7.07		Left upper limb fat, kg	0.71 ± 0.38	0.47 ± 0.15
	Weight, kg	57.3 ± 10.67	69.5 ± 9.74		Right upper limb fat, kg	0.73 ± 0.41	0.53 ± 0.22
	BMI	22.0 ± 3.75	23.3 ± 2.54		Trunk fat, kg	8.11 ± 4.51	6.07 ± 2.77
	WHR	0.83 ± 0.081	0.86 ± 0.031	Heart and blood vessels	EF, %	0.404 ± 0.034	0.389 ± 0.032
	Age, years	37.2 ± 11.85	35.6 ± 10.75		SBP, mmHg	108.0 ± 12.91	115.8 ± 8.35
Physical fitness	Grip strength, kg	25.3 ± 4.77	41.0 ± 6.82		DBP, mmHg	67.6 ± 8.04	73.0 ± 6.33
	Vital capacity, ml	2496.9 ± 545.89	3851.1 ± 857.63		PP, mmHg	40.4 ± 6.67	42.8 ± 6.94
	Reaction time, sec	0.56 ± 0.128	0.49 ± 0.106		AI, %	0.68 ± 0.150	0.55 ± 0.146
	Sit-and-reach, cm	11.7 ± 8.94	9.7 ± 9.18		Cap, mmHg	95.3 ± 15.34	96.1 ± 10.74
	Balance ability, s	48.5 ± 54.77	35.4 ± 29.81		Left BAPWV, m/s	11.98 ± 1.41	12.11 ± 1.13
Body composition	Body fat, %	27 ± 6.5	17 ± 4.5		Right BAPWV, m/s	12.84 ± 1.72	12.83 ± 1.29
	Water, %	51.7 ± 4.4	58.1 ± 3.4		Left lower limb ABI	1.19 ± 0.066	1.18 ± 0.065
	Muscle, %	17.6 ± 1.9	20.4 ± 1.1		Right lower limb ABI	1.17 ± 0.058	1.19 ± 0.070
	Inorganic salt, %	4.1 ± 0.33	4.2 ± 0.26		SEVR	1.21 ± 0.17	1.30 ± 0.18
	Left lower limb muscle, kg	6.45 ± 0.81	9.67 ± 1.67	Bone density	STI	96 ± 15.42	105 ± 17.47
	Right lower limb muscle, kg	6.53 ± 0.74	9.85 ± 1.69		BUA	45.98 ± 4.42	48.69 ± 6.03
	Left upper limb muscle, kg	1.65 ± 0.32	2.41 ± 0.61		SOS	1582 ± 27.14	1595 ± 30.10
	Right upper limb muscle, kg	1.76 ± 0.34	2.55 ± 0.64		T-value	−0.5 ± 0.83	−0.04 ± 0.91
	Trunk muscle, kg	22.25 ± 1.89	29.47 ± 2.94		Z-value	−0.02 ± 0.99	0.38 ± 1.18
	Left lower limb fat, kg	3.38 ± 1.12	2.34 ± 0.63		STI/expected value of peers	0.99 ± 0.156	1.04 ± 0.184
	Right lower limb fat, kg	3.43 ± 1.14	2.39 ± 0.69				

BMI—body mass index; WHR—waist-to-hip ratio; reaction time—the time measured by the human eye from seeing different signal lights to triggering the button by hand, which can express human agility; sit-and-reach—used to measure human flexibility; balance ability—time to stand on one foot with eyes closed; EF—ejection fraction; SBP—systolic blood pressure; DBP—diastolic blood pressure; PP—pulse pressure; AI—augmentation index; Cap—central arterial pressure; BAPWV—brachial–ankle pulse wave velocity; ABI—ankle/brachial index; SEVR—radial artery subendocardial viability ratio; STI—bone strength index; BUA—ultrasonic broadband attenuation; SOS—ultrasonic conduction sound velocity; T-value—results of bone density compared to normal young people of the same sex; Z-value—results of bone density compared to people of the same sex and age. Body composition data were measured by the bioelectrical impedance analysis method. The bone density data were measured by the ultrasonic transmission method.

## Data Availability

Not applicable.
